# A scenario based approach to optimizing cost-effectiveness of physician-staffed Helicopter Emergency Medical Services compared to ground-based Emergency Medical Services in Finland

**DOI:** 10.1186/s13049-024-01231-z

**Published:** 2024-07-02

**Authors:** Axel Ackermann, Jukka Pappinen, Jouni Nurmi, Hilla Nordquist, Anssi Saviluoto, Santtu Mannila, Simo Mäkelä, Paulus Torkki

**Affiliations:** 1 Department of Public Health, Faculty of Medicine, University of Helsinki, P.O. BOX 00020, Helsingin Yliopisto, Helsinki, 00014 Finland; 2 Helsinki University Hospital and University of Helsinki, Acute, Medical Helicopter FinnHEMS 10, Vesikuja 9, Vantaa, 01530 Finland; 3 FinnHEMS Oy, c/o Avia Pilot, Lentäjäntie 3, Vantaa, 01530 Finland; 4 South-Eastern Finland University of Applied Sciences, Department of Healthcare and Emergency care, Pääskysentie 1, Kotka, 48220 Finland; 5 Emergency Medicine and Services, University of Helsinki and Helsinki University Hospital, Helsinki, Finland; 6 Copterpoint Oy, Vähäniityntie 18B, Helsinki, 00570 Finland; 7 Data Science, University of Helsinki and Helsinki University Hospital, Helsinki, Finland

**Keywords:** Helicopter Emergency Medical Services, Air ambulances, Cost-effectiveness, ICER, Prehospital care, Optimization

## Abstract

**Objectives:**

Since Helicopter Emergency Medical Services (HEMS) is an expensive resource in terms of unit price compared to ground-based Emergency Medical Service (EMS), it is important to further investigate which methods would allow for the optimization of these services. The aim of this study was to evaluate the cost-effectiveness of physician-staffed HEMS compared to ground-based EMS in developed scenarios with improvements in triage, aviation performance, and the inclusion of ischemic stroke patients.

**Methods:**

Incremental cost-effectiveness ratio (ICER) was assessed by comparing health outcomes and costs of HEMS versus ground-based EMS across six different scenarios. Estimated 30-day mortality and quality-adjusted life years (QALYs) were used to measure health benefits. Quality-of-Life (QoL) was assessed with EuroQoL instrument, and a one-way sensitivity analysis was carried out across different patient groups. Survival estimates were evaluated from the national FinnHEMS database, with cost analysis based on the most recent financial reports.

**Results:**

The best outcome was achieved in Scenario 3.1 which included a reduction in over-alerts, aviation performance enhancement, and assessment of ischemic stroke patients. This scenario yielded 1077.07–1436.09 additional QALYs with an ICER of 33,703-44,937 €/QALY. This represented a 27.72% increase in the additional QALYs and a 21.05% reduction in the ICER compared to the current practice.

**Conclusions:**

The cost-effectiveness of HEMS can be highly improved by adding stroke patients into the dispatch criteria, as the overall costs are fixed, and the cost-effectiveness is determined based on the utilization rate of capacity.

**Supplementary Information:**

The online version contains supplementary material available at 10.1186/s13049-024-01231-z.

## Introduction

Helicopter Emergency Medical Services (HEMS) are an integral component of many healthcare systems globally, providing rapid and advanced prehospital care for critically ill patients. While these services bridge the medical gap in rural areas and reduce transport times to hospitals, their high operational costs have initiated debates about their cost-effectiveness [1–3]. In Finland, HEMS forms a crucial part of the Emergency Medical Services (EMS) and understanding the intricacies of their deployment and effectiveness is paramount. This is underscored by Finland’s notable 66% rate of over-alerts, attributed to varied reasons including imprecise risk assessments and logistical challenges [4]. Over-alerts occur when after receiving the task, the HEMS unit decides to cancel the task which can happen either before leaving the base, on the way to the patient, or at the patient.

Across many countries, the decision to dispatch a HEMS unit relies on predetermined alert criteria and expert assessment, which considers both the patient’s potential medical needs and logistical factors. In Finland, the protocol for activating HEMS mirrors that for other EMS: an emergency center’s information system evaluates the potential risk based on a set of queries, determining the response type. However, the high cancellation rate, often post-initial paramedic assessment, indicates the system’s imprecision. There is a strong consensus that patients with severe trauma benefit from advanced prehospital care, and thus, some HEMS units globally specialize in this domain [5]. However, considering the limited number of severe trauma patients and the potential for significant benefits among other patient groups, most European HEMS units extend their services to other patient groups as well, such as critically ill and stroke patients. Finnish HEMS, however, predominantly serves trauma, out-of-hospital cardiac arrest (OHCA), and unconscious patients, with stroke patients only treated in specific cases requiring advanced interventions [4].

A recent evaluation of the cost-effectiveness of Finnish HEMS compared to ground-based EMS displayed an incremental cost-effectiveness ratio (ICER) between 43,688–56,918 € per quality-adjusted life year and found HEMS to be cost-effective from a societal willingness to pay perspective [6]. Given the higher cost of HEMS compared to ground-based EMS, and the need to accordingly use public funds among different services, exploring ways to make these services more efficient is crucial. To date, there has been limited research on HEMS optimization, and it has been difficult to find studies specifically addressing the optimization point of view. Exploring broader HEMS benefits is crucial, and this investigation could potentially have impacts extending beyond Finland, influencing HEMS practices and policy decisions in other parts of the world.

The purpose of this study was to assess the cost-effectiveness of Finnish HEMS in developed scenarios with improvements in triage, aviation performance, and the inclusion of stroke patients. The study compared the effectiveness of HEMS units staffed with physicians to ground-based EMS with Advanced Life Support (ALS), using the ICER. Additionally, the study evaluated 30-day mortality.

## Methods

This was a health economic study conducted as part of a project initiated by the Prime Minister’s Office of Finland [7]. Data was obtained from the FinnHEMS database (FHDB), which included all HEMS missions from January 1, 2012, to September 9, 2019. The FHDB aligns with international guidelines for physician-staffed prehospital services [8–10]. In 2022, it contained data on around 150,000 tasks and 50,000 patients, positioning it as one of the largest databases for prehospital critical care. The FHDB has been described in detail in 2020 [4].

### Setting

The national HEMS system was organized by five university hospitals during the study period, and FinnHEMS Ltd., a state owned, publicly funded non-profit company. FinnHEMS Ltd. is responsible for providing and organizing flight services, base infrastructure, HEMS ground units and IT services. The medical staff (physicians) medical devices and other related medical services are provided and publicly funded by the university hospitals.

The HEMS units are staffed on a 24/7 basis and are equipped to fly under instrument flight rules and perform nighttime operations using night vision goggles. Each unit comprises a physician, a HEMS crew member, and a pilot. The unit in the hospital district of Lapland stands an exception, fielding two advanced-level paramedics and two pilots. Each base is equipped with rapid response vehicles for short-range missions or instances where weather conditions prohibit airborne operations.

ALS units are staffed with two paramedics, and at least one of them must hold a bachelor’s degree in emergency care (4 years of training at a University of Applied Sciences) or a bachelor’s degree in nursing, complemented by additional advanced-level prehospital specialization.

During the study period, six HEMS bases were operational, and the number of bases is set to increase to a total of eight bases in fall 2024. Our study was conducted using the model of eight bases. The operational dynamics of the Finnish HEMS system has been comprehensively described in prior research [4].

### Introducing the patient groups

The patient demographics of the Finnish HEMS system has been assessed in a recent study [11]. We identified the primary groups of patients that comprise the majority of those assisted by HEMS, with evidence suggesting these groups either gain medical or logistical support from HEMS. This was guided by their notable prevalence in the FHDB, corroborated by our literature review and own findings. Consequently, the service need was evaluated for the following patient groups: severe trauma, OHCA, those requiring anaesthesia and intubation, ischemic stroke, and other patients. Stroke patients were selected as a new group as they represent a significant group in healthcare systems akin to Finland’s [5]. Given Finland’s expansive geography and the centralization of stroke care at university hospitals, alongside HEMS’s low utilization rate (time a HEMS unit spends on a task, from receiving it to returning to base for the next task, divided by a year), their inclusion emerges as a prudent and feasible choice.

### Modelling method

In the modelling, a service needs assessment forecast was created, integrating data from completed HEMS missions, the entirety of EMS missions coordinated by the emergency center, specific risk classifications on a municipal level, road network information, and population statistics. The modelling incorporated findings that centralizing advanced prehospital care with specialized teams lowers patient mortality, informed by literature and HEMS experts [12, 13].

Given the recent publication of a systematic review on the effectiveness of HEMS [5], we chose not to conduct another comprehensive systematic review within our model. Instead, we utilized a rapid review strategy [14] and updated our data search criteria to reflect the most recent information. Search terms from the previous review were employed in the PubMed and EMBASE databases, carried out on November 2, 2021. All articles cited in the previous review were integrated without necessitating a new evaluation. The search criteria are provided in Appendix 1.

Our search did not uncover any new original publications that met our search criteria following the published systematic review. As a result, we decided not to reiterate the data collection and reporting process. Instead, we focused on a comprehensive review of the articles, placing particular emphasis on determining the relevance of these studies to the context of Finnish HEMS system including 1) the specialization level of physicians, 2) number of tasks per unit and per physician, 3) the quality of care provided by other EMS, and 4) other central factors affecting applicability, such as geography, demographics and health care systems.

The service needs assessment forecast was developed utilizing a grid database, where Finland is divided into 1 × 1 km size squares. For each 1 km2 square, the following background variables were defined: 1) risk category classification [15, 16], 2) location in relation to Finnish road network (road connections, length of roads in the grid, and highest speed limit 80 km/h) [17], 3) municipality, hospital district, and university hospital area, 4) permanently resident population, 5) number of EMS missions (including other than HEMS) classified by dispatch urgency and code, 6) estimated response time of a ground-unit to the grid’s center, using historical response times of missions by the same urgency, applying the Inverse Distance Weighting (IDW) interpolation method [18], 7) Driving time and distance from nearest base to closest road point to the grid center, and from this point to University hospital or large-scale emergency hospital [19], and 8) flight time and distance from bases to the grid center, and from this point to the nearest university hospital or large-scale emergency hospital by average 220 km/h airspeed based on information received from FinnHEMS. The service need forecast was evaluated for each patient group.

The prevalence of OHCA patients was first determined for the entire population using research data [20], after which the data was distributed to each hospital district and first aid risk category, proportional to the workload managed by the emergency centers. Severe trauma patients’ incidence rates were predicted based on data from emergency centers task codes. For each task code related to trauma, we calculated the probability for the patient being transported to the hospital with HEMS. The need for anaesthesia and intubation was a more complex phenomenon to predict, as the need can arise from several reasons, all of which cannot be precisely modelled [4, 21]. The “other” patient group was determined using the FHDB of patients not fitting into the main patient groups and who were hospital-admitted by a HEMS physician. For the anaesthesia and intubation group, as well as the “other” patient group, we used extrapolation based on the completed tasks recorded in the FHDB. This involved determining the number of risk-categorized patients for each inhabitant of the hospital district around each base and then extending these figures to the surrounding hospital districts, proportionally to the population size of each district. Stroke patients’ geographical distribution was modelled according to research data [22–24] and information from the emergency center using the task code for acute stroke.

The modeling included the availability of aviation service, which represents the probability of a HEMS helicopter completing a mission from dispatch to return. This factor is crucial in determining both service availability and patient outcomes. Weather conditions, due to their substantial annual variations, play a significant role in helicopter use, dictating requirements for the helicopter fleet, infrastructure, and base network. The probability of helicopter deployment was estimated using weather data from 10 years for each square kilometer in the grid [25]. The base locations and helicopter types (currently EC135 and EC145 in Finland) further influence service availability, especially on a regional level, under the purview of national and EASA regulations. These helicopter models define operational ranges (263–353 NM), which can be extended by increasing flight altitude, but a standard altitude of 2000 ft is commonly used to prevent icing. Internal HEMS operational factors affecting service availability, such as crew availability and helicopters’ technical problems accounted for a negligible impact of only 0,07%, according to FHDB data, hence were disregarded in the model.

### Estimating health benefits

Health benefits were measured using the estimated 30-day mortality rate, which is a commonly used and universally accepted metric in medical research for assessing the impact on critical patient groups. For stroke patients, health benefits were assessed based on recovery to good functional capacity, defined by modified Ranking Scale (mRS; values of 0–2).

The modelling suggests that patients who stand to benefit most from HEMS intervention are those reached within 30 min after an alarm is activated. However, pinpointing an exact timeframe to determine treatment benefits remains challenging. For instance, the overall mortality rate among injured patients is not solely attributed to response delays. Yet, observational data suggests that aiming for a 30-min response aligns with recommended practices, like those from the UK’s National Institute for Health and Care Excellence guidelines, which advise having anaesthesia ready within 45 min post-incident [26]. The additional benefit produced by HEMS was estimated for each patient group based on the literature [21, 27–30].

### Estimating patient survival

Patient survival was assessed based on the methodology of a recent study [6]. The survival analysis was based on data from the FHDB, covering all HEMS-treated patients from January 1, 2012, to September 8, 2019 [31]. Survival rates were calculated until 3 years, then aligned it with general population life expectancy. The median age of patients treated by Finnish HEMS was 57.7 years, with a breakdown of 63% male, 35% female, and 2% gender unspecified, and therefore, we used 23 years remaining life expectancy. For ischemic stroke patients, the mean age was 73.1 years [32], and we used 8 years for remaining life expectancy.

### Estimating quality of life

We assessed the impact of HEMS intervention on patients’ QoL 3 years post-intervention, utilizing the EuroQoL EQ-5D scale. This scale is a prominent instrument for evaluating health-related QoL (HRQoL) across diverse patient demographics [33]. It enables the comparison of health outcomes to an ideal state of health [34]. Literature suggests that for the patient groups involved in this study, expected EuroQoL index values post-intervention range from 0.6 to 0.8 [35–42]. Nonetheless, specific index values for these patient groups are not well-documented, and results tend to fluctuate due to patient group heterogeneity and study limitations. This inconsistency precludes a direct application of QoL measures to FinnHEMS patients. To address potential outcome variability and ensure the reliability of our conclusions, we executed a sensitivity analysis focusing on this QALY-index spectrum.

### Cost assessment

The financial structure of FinnHEMS Ltd. was recently evaluated [6], informed by the company’s 2021 financial statements. Operational expenses for the six bases which were active in 2022 was approximately €30 million. The activation of two additional bases is expected to increase the total operational costs to €40 million annually for the full network of eight bases. In addition, medical service costs, which cover physician salaries, medical equipment, and other related services, add an estimated €8 million per year, bringing the projected annual expenditure for eight bases to €48 million.

Fixed costs constituted 93% of the total costs, attributed to the contingent nature of operations which require staff readiness, regardless of actual service deployment. Variable costs (7%) were linked to flight activity and included maintenance of equipment and fuel costs. Additional costs have been identified in different scenarios where aviation operations were enhanced. This included the establishment of the Point in Space (PiNS) navigation network and the implementation of a new, more costly helicopter fleet with the Full Ice Protection System (FIPS), which enabled flight operations under severe weather conditions.

### Introducing scenarios

The scenarios were developed with guidance from the Finnish Prime Ministry’s steering committee, in collaboration with experts, assimilating practices from global HEMS systems, and a comprehensive review of literature. Optimization of HEMS in different scenarios was approached with improvements in triage, level of medical care and aviation performance (Table 1). In every scenario, HEMS was modelled to achieve the same effectiveness, with the closest HEMS unit being dispatched and the patient transported to the closest university hospital, or in certain instances, to the closest large-scale emergency hospital, without consideration for administrative (such as hospital district) boundaries. Medical units not part of the HEMS system were excluded from the modelling. The Helsinki city area was disregarded from the modelling due to its established mobile physician-staffed intensive care operations. Service needs or assignments in Åland were also not considered in the model.

**Table 1:** Overview of HEMS optimization scenarios

	**Scenario 1**	**Scenario 2**	**Scenario 2.1**	**Scenario 3**	**Scenario 3.1**	**Scenario 3.2**
**Over-Alerts Reduced**	Yes (30%)	No	No	Yes (30%)	Yes (30%)	Yes (30%)
**Aviation Expansion**	No	Yes	Yes	No	Yes	Yes
**FIPS for Extreme Weather**	No	No	Yes	No	No	Yes
**Stroke Patients**	No	No	No	Included	Included	Included
**Cost Drivers**	No additional costs	PiNS network and cloud penetration	FIPS, PiNS network and cloud penetration	No additional costs	PiNS network and cloud penetration	FIPS, PiNS network and cloud penetration

*FIPS* Full Ice Protection System, *PiNS* Point-in-Space

In Scenario 1, there was a decrease in the number of tasks that ultimately led to cancellations. Only 30% of over-alerts (currently 66%) occurred for patients benefiting from the service. Enhancements could be achieved through better expertise in risk assessment, the centralization of HEMS needs assessment into a single, nationwide unit, and improved patient identification protocols that utilize previous health records. Additionally, the adoption of technical innovations, such as real-time video live feed from the emergency site, could enhance the precision of dispatch decisions.

For Scenario 2, the availability of aviation services was expanded while the task selection remained unaltered. This setup incorporated the 2023 updates to night visual flight rules (VFR) weather minimum regulations [43–45]. A comprehensive Point-in-Space (PinS) network for HEMS across Finland was developed, which is based on satellite navigation and is used for accurate and safe helicopter approach and departure procedures in limited visibility conditions [46]. Its cost estimates were derived from data provided by the Norwegian Air Ambulance Foundation which offer a useful benchmark for Finland. Furthermore, a radial 360° low-altitude flight route was designed around each base, enabling cloud penetration in the surrounding sectors. While it is challenging to quantify the cost implications of this feature, it is expected to be substantially less expensive than the PinS network. Thus, its associated costs are assumed to be subsumed under the PinS network expenditures.

In Scenario 2.1, we considered the implications of extreme weather conditions. This requires new, more robust helicopter fleet equipped with Full Ice Protection System (FIPS). Based on expert input, implementing FIPS is estimated to increase equipment and maintenance costs by 15–40%.

Scenario 3 builds upon Scenario 1 by including ischemic stroke patients within the service scope. This inclusion relied on using a clinical status screening tool for identifying large vessel occlusion (LVO) to differentiate between patients requiring thrombectomy and those suitable for thrombolysis. Acknowledging the tool’s limited accuracy in distinguishing between haemorrhagic strokes, stroke mimics, and true LVO cases, the model factored in that 1.9 patients were transported to the nearest university hospital for every thrombectomy performed [47] and 2.6 patients to the nearest extensive emergency hospital for every thrombolysis conducted [48]. Given that hospitals offering thrombectomy are generally farther from the patients, accurate LVO identification is critical to minimizing unnecessary thrombectomy delays. Importantly, the inclusion of stroke patients into the dispatch criteria was modelled to not detrimentally impact the service provided to other patient groups, attributable to a reduction in over-alarming. Consistently, aviation operations adhered to existing regulations and operational capacities.

Scenario 3.1 integrated the approaches of Scenarios 1 and 2, adding ischemic stroke patients to the service to enhance accessibility and streamline both task allocation and aviation operations.

Scenario 3.2 combined the components of Scenarios 1, 2, 2.1 and included ischemic stroke patients.

The results were obtained over a patient’s remaining life expectancy of 23 years, and 8 years for stroke patients and included a sensitivity analysis on the QALY-indexes ranging from 0.6–0.8. The differential ICER across the scenarios provides a comprehensive perspective on the potential economic value of HEMS in different contexts and under varied assumptions.

## Results

### Estimated health benefits

The modelling compared the interventions of advanced HEMS physician grade emergency treatment and ALS ground-based EMS. Severe trauma patients had 5,3% smaller absolute risk for 30-day mortality with a HEMS intervention and a NNT of 18,8. For OHCA patients the risk for absolute mortality was reduced 1,3% and NNT after successful resuscitation was 76,0. NNT for all OHCA tasks was 213,4. Patients in the need for anaesthesia and intubation (excluding trauma and OHCA patients) had 3,7% reduced absolute risk for 30-day mortality and NNT of 27,4. For stroke patients, the modelling compared helicopter transport with ground-ambulance transport when time saving is achievable. Stroke patients in the reach for mechanical thrombectomy had 5,1% reduced absolute risk for reduced functional capacity (mRs scale) per 1 h of time saved. Stroke patients in the reach for thrombolysis had 4,7–22% reduced absolute risk for non-exellent recovery (mRS > 1) depending on the delay (22% in 90 min, 11% in 180 min, 7.1% in 270 min, 4.7% in 360 min). Patients classified as ‘other’ were estimated to have an NNT of 25, informed by FHDB and expert judgment, reflecting the similar intensity of life support to trauma and intubation group. Due to the heterogeneity of patient groups [11], it is impossible to obtain exact information from this group.

### Cost-effectiveness

Our results (Table 2) showed that the cost-effectiveness of HEMS was best improved by including stroke patients in the dispatch criteria (Scenarios 3, 3.1 and 3.2). Every year, the loss of functional capacity of 37.5 stroke patients was prevented by shortening the transport time to thrombolysis or thrombectomy. This obtained approximately 180.1–240.1 additional QALYs annually with HEMS operations. The best outcome was achieved in Scenario 3.1 which included a reduction in over-alerts, aviation performance enhancement and the assessment of ischemic stroke patients, with total annual costs at €48,4 M. In this scenario 1077.07–1436.09 additional QALYs were achieved with an ICER of 33,703–44,937 €/QALY. This was a 27.72% increase in the additional QALYs and a 21.05% reduction in the ICER compared to the current practice.

**Table 2:** Results of HEMS scenarios: Comparative analysis of health outcomes, costs, and ICER

	**Number of alarms**	**Prevented 30-day mortality**	**Stroke patients with good functional recovery**	**Total annual costs**	**QALYs**	**ICER**
**Scenario 1**	5940	69.8	-	48,0 M€	864.87–1153.17	41 625–55 499 €/QALY
**Scenario 2**	15,763	70.2	-	48,4 M€	869.39–1159.19	41 753–55 671 €/QALY
**Scenario 2.1**	15,763	73.9	-	50,2–53,1 M€	914.98–1219.98	41 126–58 063 €/QALY
**Scenario 3**	5940	69.8	37.5	48,0 M€	1382.58–1843.44	26 038–34 718 €/QALY
**Scenario 3.1**	5940	72.1	37.5	48,4 M€	1414.70–1886.27	25 659–34 212 €/QALY
**Scenario 3.2**	5940	76.1	37.5	50,2–53,1 M€	1469.90–1959.87	25 600–36 143 €/QALY

*QALYs* Quality-adjusted life years, *ICER* Incremental cost-effectiveness ratio

In Scenarios 1 and 2, costing annually €48,0 M and €48,4 M respectively, the cost-effectiveness did not change substantially. Therefore, these alternatives must be evaluated from other perspectives, such as from the perspective of accessibility, equality, or other operational principles. In Scenario 2.1 with total annual costs at €50,2–53,1 M, the cost-effectiveness decreased a little primarily because the de-icing functionality practically required a more robust helicopter fleet and thus increased the costs.

## Discussion

This study provides a thorough assessment on the optimization of the cost-effectiveness of Finnish HEMS in developed scenarios, concentrating on the prevention of 30-day mortality and stroke patients’ recovery to good functional health. It fills an important gap in existing literature, representing the first in-depth evaluation on the cost-effectiveness optimization of HEMS with multiple patient groups. By integrating stroke patients into the HEMS dispatch criteria, the value of these services can be significantly improved, as reflected by the increase in QALYs and the reduction in ICER in Scenario 3.1. The outcome highlights the potential for targeted improvements in patient triage and service deployment to gain economic benefits and improved patient outcomes.

Considering that the majority of costs are fixed and determined by the number of bases and equipment, optimizing the utilization rate is a key driver for enhancing cost-effectiveness, as HEMS can distribute the fixed costs across a wider range of patients. Currently, in Finland, the mean utilization rate is 17.3% (2018). Since politicians decide the scope and scale of operations, an increase in the utilization, which they can influence, could make the operations significantly cheaper. On the other hand, the impact of variable costs on the overall cost-effectiveness is marginal and is predominantly a matter of fine-tuning rather than a solution for economic improvement.

Another perspective for improving cost-effectiveness could lie in potential cooperation with other national actors, especially regarding future helicopter acquisitions. A larger number of the same helicopter type might enable fewer helicopters needed for maintenance purposes. It’s also worth considering the broader role of HEMS in implementing the best possible care within the current operational framework. As HEMS crews tend to have a wide range of competencies and experience [49], efforts in consultations [50], training activities [51, 52], and standardizing the care provided across different regions [53] could be future development areas that might impact the comprehensive care pathways for stroke patients.

The screening tool identified patients with LVO relatively well, although the development of criteria to identify thrombolysis based on time frames and functional capacity would likely increase these numbers to align with thrombectomy rates. However, due to the absence of validated instruments or supporting data, this study adopts a more conservative estimate. A significant advancement in this direction could be the development of CT imaging technology compact enough for helicopter use [54]. This innovation could transform stroke care by enabling the initiation of thrombolysis treatment directly at the patient location, dramatically reducing the time to treatment for stroke patients and potentially improving outcomes.

Looking ahead, the anticipated pruning of the Finnish emergency network emphasizes the critical need to expand HEMS’s patient criteria, ensuring nationwide comprehensive care, especially in remote areas, as emergency services undergo strategic restructuring.

It is important to note that preventing mortality is only a single viewpoint of HEMS’s contributions. HEMS also provides care that can result in shorter hospitalizations and quicker recovery for patients who might survive without EMS support or air transport. Research on this topic is still quite scarce and more detailed comparative studies are needed [55]. On the other hand, some patients may survive but continue living with severe disability. Currently, the treatment outcomes including QoL metrics are not systematically recorded in Finland. We recommend that QoL metrics be routinely documented in HEMS operations, allowing for a more precise assessment of real-world effectiveness and international comparisons of the outcomes for different patient groups.

Our analysis did not include a discount rate, given the ongoing debate about its suitability for evaluating health benefits [56, 57]. The incorporation of a sensitivity analysis on QALYs and the use of estimated values over exact numbers in our approach further reduce the need for a discount rate.

There are limitations in our model. The QALYs are calculated over a patient’s lifetime, whereas costs are based solely on annual HEMS expenditures. Survivors may incur further costs in hospital treatment and rehabilitation, but our QALY-index sensitivity analysis suggests a favorable health status, indicating that these expenses may not substantially affect the broader economic perspective.

Data from the FHDB, with potential inconsistencies and incomplete records due to manual entry by physicians, also presents limitations [4]. The HRQoL indexes used do not provide precise values for different patient groups, as literature lacks detailed information. The cost data for ground-based units within the Finnish system were not available, presenting a limitation in our cost assessment.

The health benefits are derived from literature [21, 27–30] and cannot be completely generalized to Finnish population. Our study included patients reachable within 30 min, but those with longer reach delays may also benefit from the service. However, the impact of delay in reaching patients in HEMS operations does not seem to have a major impact on mortality [58]. Furthermore, the estimated number of patients is based on a statistical model, as the two additional bases do not yet exist, and the current patient selection does not entirely reflect the actual situation.

## Conclusions

This study provides a broad assessment on the optimization of cost-effectiveness of Finnish HEMS at a national level, by examining the ICER between physician-staffed HEMS and ground-based EMS. The results show that the cost-effectiveness of HEMS can be highly improved by including stroke patients in the dispatch criteria, as most of the costs are fixed and costs are determined based on the utilization of capacity.

### Supplementary Information

Supplementary Material 1.

## Data Availability

The datasets used and/or analysed during the current study available from the corresponding author on reasonable request.

## Abbreviations

HEMS: Helicopter emergency medical services
EMS: Emergency medical services
ICER: Incremental cost-effectiveness ratio
OHCA: Out-of-hospital cardiac arrest
HRQoL: Health-related quality of life
QoL: Quality of life
QALY: Quality-adjusted life year
FHDB: FinnHEMS database
EQ-5D: European quality of life five dimension
PiNS: Point-in-Space network
FIPS: Full ice protection system
