# Supplementary material for: A scenario based approach to optimizing cost-effectiveness of physician-staffed Helicopter Emergency Medical Services compared to ground-based Emergency Medical Services in Finland
Source: Scand J Trauma Resusc Emerg Med. 2024 Jul 2;32:60. doi: 10.1186/s13049-024-01231-z (PMC11221128; doi:10.1186/s13049-024-01231-z)
Supplement: Supplementary file 1 — Supplementary Material 1. [file 13049_2024_1231_MOESM1_ESM.docx]

Appendix 1

1. “Air Ambulances”[Mesh] OR ((“Aviation”[Mesh] OR “Aircraft”[Mesh] OR “Aerospace Medicine”[Mesh]) AND (“Ambulances”[Mesh] OR “Emergency Medical Services”[Mesh] OR “Emergency treat- ment”[Mesh] OR “Transportation of patients”[Mesh]))
2. "Prospective Studies"[Mesh]) OR "Controlled Clinical Trials as Topic"[Mesh]) OR "Controlled Clinical Trial" [Publication Type]) OR "Controlled Before-After Studies"[Mesh]
3. #1 AND #2
4. Helicopter* AND (Emergency* OR Ambulance* OR “transportation of patients")
5. controlled OR prospective* OR random*
6. #4 AND #5 7. #3 OR #6
